# Supplementary material for: Multi‐dataset identification of innovative feature genes and molecular mechanisms in keratoconus
Source: J Cell Mol Med. 2024 Sep 19;28(18):e70079. doi: 10.1111/jcmm.70079 (PMC11412914; doi:10.1111/jcmm.70079)
Supplement: Supplementary file 2 — Material S2. [file JCMM-28-e70079-s002.zip › 7.GSEA analysis/5.RNF217-AS1/KEGG_PARKINSONS_DISEASE.html]

Details for gene set KEGG\_PARKINSONS\_DISEASE[GSEA]

|  || Dataset | RNF217-AS1.group.cls#h\_versus\_l |
| Phenotype | group.cls#h\_versus\_l |
| Upregulated in class | l |
| GeneSet | KEGG\_PARKINSONS\_DISEASE |
| Enrichment Score (ES) | -0.31623995 |
| Normalized Enrichment Score (NES) | -1.333781 |
| Nominal p-value | 0.17979798 |
| FDR q-value | 0.3613652 |
| FWER p-Value | 0.931 |
Table: GSEA Results Summary

  

Fig 1: Enrichment plot: KEGG\_PARKINSONS\_DISEASE      
 Profile of the Running ES Score & Positions of GeneSet Members on the Rank Ordered List

  

| SYMBOL | TITLE | RANK IN GENE LIST | RANK METRIC SCORE | RUNNING ES | CORE ENRICHMENT || 1 | SLC25A31 | na | 1638 | 0.379 | 0.0125 | No |
| 2 | COX7B2 | na | 6236 | 0.176 | -0.0924 | No |
| 3 | TH | na | 7858 | 0.131 | -0.1185 | No |
| 4 | CASP3 | na | 9912 | 0.088 | -0.1639 | No |
| 5 | COX4I2 | na | 11142 | 0.068 | -0.1886 | No |
| 6 | COX7A2L | na | 11823 | 0.058 | -0.1991 | No |
| 7 | UBE2G1 | na | 11990 | 0.055 | -0.1952 | No |
| 8 | COX6B2 | na | 12168 | 0.053 | -0.1919 | No |
| 9 | VDAC2 | na | 12701 | 0.046 | -0.2000 | No |
| 10 | NDUFA10 | na | 14013 | 0.031 | -0.2330 | No |
| 11 | NDUFB5 | na | 14086 | 0.030 | -0.2304 | No |
| 12 | APAF1 | na | 14427 | 0.026 | -0.2362 | No |
| 13 | UBE2L3 | na | 14433 | 0.025 | -0.2323 | No |
| 14 | COX7B | na | 14914 | 0.020 | -0.2430 | No |
| 15 | SLC18A2 | na | 15277 | 0.016 | -0.2509 | No |
| 16 | COX6C | na | 15464 | 0.014 | -0.2540 | No |
| 17 | NDUFV1 | na | 15508 | 0.014 | -0.2530 | No |
| 18 | NDUFS6 | na | 15562 | 0.013 | -0.2524 | No |
| 19 | NDUFA1 | na | 15926 | 0.010 | -0.2614 | No |
| 20 | NDUFS4 | na | 16079 | 0.008 | -0.2646 | No |
| 21 | NDUFS1 | na | 16420 | 0.004 | -0.2737 | No |
| 22 | NDUFB1 | na | 16574 | 0.003 | -0.2776 | No |
| 23 | COX6B1 | na | 16711 | 0.002 | -0.2813 | No |
| 24 | COX7C | na | 16867 | -0.000 | -0.2858 | No |
| 25 | NDUFB3 | na | 16984 | -0.001 | -0.2889 | No |
| 26 | COX5B | na | 17009 | -0.002 | -0.2893 | No |
| 27 | SDHD | na | 17106 | -0.003 | -0.2917 | No |
| 28 | UQCRC2 | na | 17167 | -0.003 | -0.2929 | No |
| 29 | UQCR11 | na | 17489 | -0.006 | -0.3012 | No |
| 30 | NDUFAB1 | na | 17544 | -0.007 | -0.3017 | No |
| 31 | NDUFC1 | na | 17617 | -0.007 | -0.3026 | No |
| 32 | NDUFS5 | na | 17643 | -0.008 | -0.3021 | No |
| 33 | COX7A2 | na | 17707 | -0.008 | -0.3027 | No |
| 34 | SDHA | na | 17733 | -0.008 | -0.3021 | No |
| 35 | NDUFA6 | na | 17794 | -0.009 | -0.3024 | No |
| 36 | NDUFB4 | na | 17944 | -0.010 | -0.3051 | No |
| 37 | NDUFS8 | na | 17948 | -0.010 | -0.3035 | No |
| 38 | COX6A1 | na | 18053 | -0.011 | -0.3048 | No |
| 39 | UQCRC1 | na | 18155 | -0.012 | -0.3058 | No |
| 40 | NDUFB9 | na | 18519 | -0.016 | -0.3138 | Yes |
| 41 | UQCRQ | na | 18543 | -0.016 | -0.3119 | Yes |
| 42 | COX8A | na | 18621 | -0.017 | -0.3115 | Yes |
| 43 | NDUFS3 | na | 18677 | -0.017 | -0.3104 | Yes |
| 44 | NDUFV3 | na | 18696 | -0.017 | -0.3082 | Yes |
| 45 | NDUFV2 | na | 18702 | -0.017 | -0.3056 | Yes |
| 46 | NDUFB8 | na | 18716 | -0.018 | -0.3032 | Yes |
| 47 | COX6A2 | na | 18729 | -0.018 | -0.3007 | Yes |
| 48 | SLC25A5 | na | 18805 | -0.018 | -0.3000 | Yes |
| 49 | NDUFA4 | na | 18841 | -0.019 | -0.2980 | Yes |
| 50 | COX5A | na | 19181 | -0.022 | -0.3044 | Yes |
| 51 | NDUFB7 | na | 19315 | -0.023 | -0.3045 | Yes |
| 52 | SLC25A6 | na | 19447 | -0.025 | -0.3044 | Yes |
| 53 | NDUFA9 | na | 19508 | -0.025 | -0.3021 | Yes |
| 54 | NDUFS7 | na | 19536 | -0.026 | -0.2989 | Yes |
| 55 | SLC25A4 | na | 19627 | -0.026 | -0.2973 | Yes |
| 56 | NDUFB10 | na | 19641 | -0.027 | -0.2935 | Yes |
| 57 | NDUFA7 | na | 19695 | -0.027 | -0.2908 | Yes |
| 58 | PINK1 | na | 19882 | -0.029 | -0.2916 | Yes |
| 59 | PPID | na | 20070 | -0.031 | -0.2921 | Yes |
| 60 | NDUFA3 | na | 20174 | -0.032 | -0.2901 | Yes |
| 61 | UBE2G2 | na | 20337 | -0.033 | -0.2895 | Yes |
| 62 | UQCRH | na | 20365 | -0.033 | -0.2850 | Yes |
| 63 | COX4I1 | na | 20433 | -0.034 | -0.2816 | Yes |
| 64 | NDUFA4L2 | na | 20479 | -0.035 | -0.2774 | Yes |
| 65 | VDAC3 | na | 20555 | -0.035 | -0.2739 | Yes |
| 66 | SLC6A3 | na | 20575 | -0.036 | -0.2689 | Yes |
| 67 | NDUFA8 | na | 20605 | -0.036 | -0.2640 | Yes |
| 68 | NDUFB2 | na | 20644 | -0.036 | -0.2594 | Yes |
| 69 | COX7A1 | na | 20777 | -0.038 | -0.2572 | Yes |
| 70 | SDHC | na | 20833 | -0.038 | -0.2528 | Yes |
| 71 | CASP9 | na | 20912 | -0.039 | -0.2489 | Yes |
| 72 | UBA1 | na | 21023 | -0.040 | -0.2457 | Yes |
| 73 | UQCRFS1 | na | 21182 | -0.042 | -0.2437 | Yes |
| 74 | NDUFA2 | na | 21309 | -0.043 | -0.2405 | Yes |
| 75 | UBE2J1 | na | 21352 | -0.044 | -0.2348 | Yes |
| 76 | UQCRB | na | 21369 | -0.044 | -0.2284 | Yes |
| 77 | VDAC1 | na | 21769 | -0.048 | -0.2323 | Yes |
| 78 | SDHB | na | 21783 | -0.048 | -0.2250 | Yes |
| 79 | NDUFB6 | na | 21949 | -0.050 | -0.2219 | Yes |
| 80 | UQCR10 | na | 21990 | -0.051 | -0.2150 | Yes |
| 81 | CYCS | na | 22416 | -0.055 | -0.2186 | Yes |
| 82 | NDUFA5 | na | 22469 | -0.056 | -0.2113 | Yes |
| 83 | UBB | na | 22618 | -0.057 | -0.2065 | Yes |
| 84 | PARK7 | na | 23240 | -0.065 | -0.2143 | Yes |
| 85 | LRRK2 | na | 23302 | -0.066 | -0.2057 | Yes |
| 86 | CYC1 | na | 23861 | -0.073 | -0.2103 | Yes |
| 87 | UBE2L6 | na | 24379 | -0.081 | -0.2124 | Yes |
| 88 | NDUFC2 | na | 24639 | -0.085 | -0.2065 | Yes |
| 89 | HTRA2 | na | 24978 | -0.090 | -0.2021 | Yes |
| 90 | UBE2J2 | na | 25127 | -0.092 | -0.1918 | Yes |
| 91 | NDUFS2 | na | 25676 | -0.101 | -0.1917 | Yes |
| 92 | UBA7 | na | 28686 | -0.168 | -0.2521 | Yes |
| 93 | SLC18A1 | na | 30173 | -0.212 | -0.2615 | Yes |
| 94 | GPR37 | na | 31767 | -0.277 | -0.2637 | Yes |
| 95 | UCHL1 | na | 32920 | -0.354 | -0.2410 | Yes |
| 96 | SNCA | na | 34069 | -0.497 | -0.1958 | Yes |
| 97 | SNCAIP | na | 34077 | -0.498 | -0.1174 | Yes |
| 98 | COX8C | na | 34671 | -0.862 | 0.0016 | Yes |
Table: GSEA details [plain text format]

  

Fig 2: KEGG\_PARKINSONS\_DISEASE      
 Blue-Pink O' Gram in the Space of the Analyzed GeneSet

  

Fig 3: KEGG\_PARKINSONS\_DISEASE: Random ES distribution      
 Gene set null distribution of ES for **KEGG\_PARKINSONS\_DISEASE**
